# SPAG6 Promotes Multiple Myeloma Through Activation of the MAPK/ERK Signaling Pathway

**DOI:** 10.3389/fphar.2025.1572621

**Published:** 2025-06-04

**Authors:** Junnan Li, Xinyu Yan, Li Ding, Jiaxiu Yin, Ping Li, Lin Liu

**Affiliations:** ^1^ Department of Hematology, The First Affiliated Hospital of Chongqing Medical University, Chongqing, China; ^2^ Department of Orthopaedics, Chongqing Hospital of Traditional Chinese Medicine, Chongqing, China

**Keywords:** SPAG6, sperm-associated antigen 6, DUSP1, dual specificity phosphatase, MAPK, mitogen-activated protein kinase, expresion, multiple myeloma

## Abstract

**Background:**

Sperm - associated antigen 6 (SPAG6), a member of the cancer/testis antigen (CTA) family, has been linked to multiple hematologic malignancies. Nevertheless, its role in multiple myeloma (MM) remains unclear.

**Methods:**

Bioinformatics, tissue specimens from plasma cell tumors, and bone marrow samples of MM patients were utilized to evaluate SPAG6 expression and to analyze its correlations with clinical features and prognosis. *In vitro*, RNA interference was applied to downregulate SPAG6 in U266 cells and upregulate it in RPMI - 8226 cells, and then its impacts on cell proliferation, apoptosis, and migration were investigated. Transcriptome sequencing data were comprehensively analyzed to elucidate the mechanism of SPAG6 in MM cells.

**Results:**

SPAG6 was positively expressed in MM cell lines, plasma cell tumor tissue specimens, and MM patient bone marrow samples. The mRNA expression of SPAG6 in MM patients was upregulated relative to the control group and was correlated with blood calcium levels, plasma cell ratio, and skeletal infiltration. *In vitro*, SPAG6 overexpression promoted cell proliferation, migration, and the resistance to apoptosis in MM cells, while down - expression had contrary effects. Mechanistic studies revealed that SPAG6 directly interacts with dual-specificity phosphatase 1 (DUSP1). Furthermore, SPAG6 was found to modulate the expression of downstream proteins in the mitogen-activated protein kinase (MAPK)/extracellular signal-regulated kinase (ERK) signaling pathway by regulating DUSP1 activity.

**Conclusion:**

Overall, this study highlights that SPAG6 may serve as a potential therapeutic target for MM by regulating DUSP1 expression to activate the MAPK/ERK signaling pathway.

## 1 Introduction

Multiple myeloma (MM), a common plasma cell malignancy, results from the malignant clonal hyperplasia of plasma cells. Clinically, it typically manifests as hyperproteinemia, osteolytic lesions, anemia, and renal impairment (Rajkumar and Kumar, 2020). Its pathogenesis is highly complex, involving intricate crosstalk between myeloma and plasma cells. Gene mutations, deletions, translocations, and other epigenetic aberrations drive MM progression (Adamik et al., 2019; Manier et al., 2017; Pawlyn et al., 2020). This complexity leads to marked heterogeneity in MM’s clinical features and prognosis. Patient survival varies greatly, from a few months to over a decade (Adamik et al., 2019; Pawlyn et al., 2020). Despite the importance of combined clinical treatments for prognosis, MM remains incurable (Berdeja et al., 2021; Burroughs García et al., 2021). Thus, identifying new biomarkers and optimizing personalized therapies are crucial for extending patients’ survival.

In recent years, studies have shown uniparental disomy (UPD) - related abnormalities are key in tumor development (Tuna et al., 2022). Sperm - associated antigen 6 (SPAG6) in the UPD region belongs to the CTAs family (Yan et al., 2015; Sapiro et al., 2000; Xu et al., 2022), it encodes microtubule - related proteins, regulating cilia/flagella polarization and immune processes (Yan et al., 2015). In hematological malignancies, researchers have discovered SPAG6 was upregulated in patients with myelodysplastic syndrome (MDS), acute myeloid leukemia (AML), and myeloproliferative neoplasms (MPN). Downregulation of SPAG6 could inhibit cell proliferation (Bao et al., 2022; Steinbach et al., 2006; Ding et al., 2022). This suggests SPAG6 affects hematological tumor cell development and could be as a potential target for hematological tumors.

**FIGURE 1:**
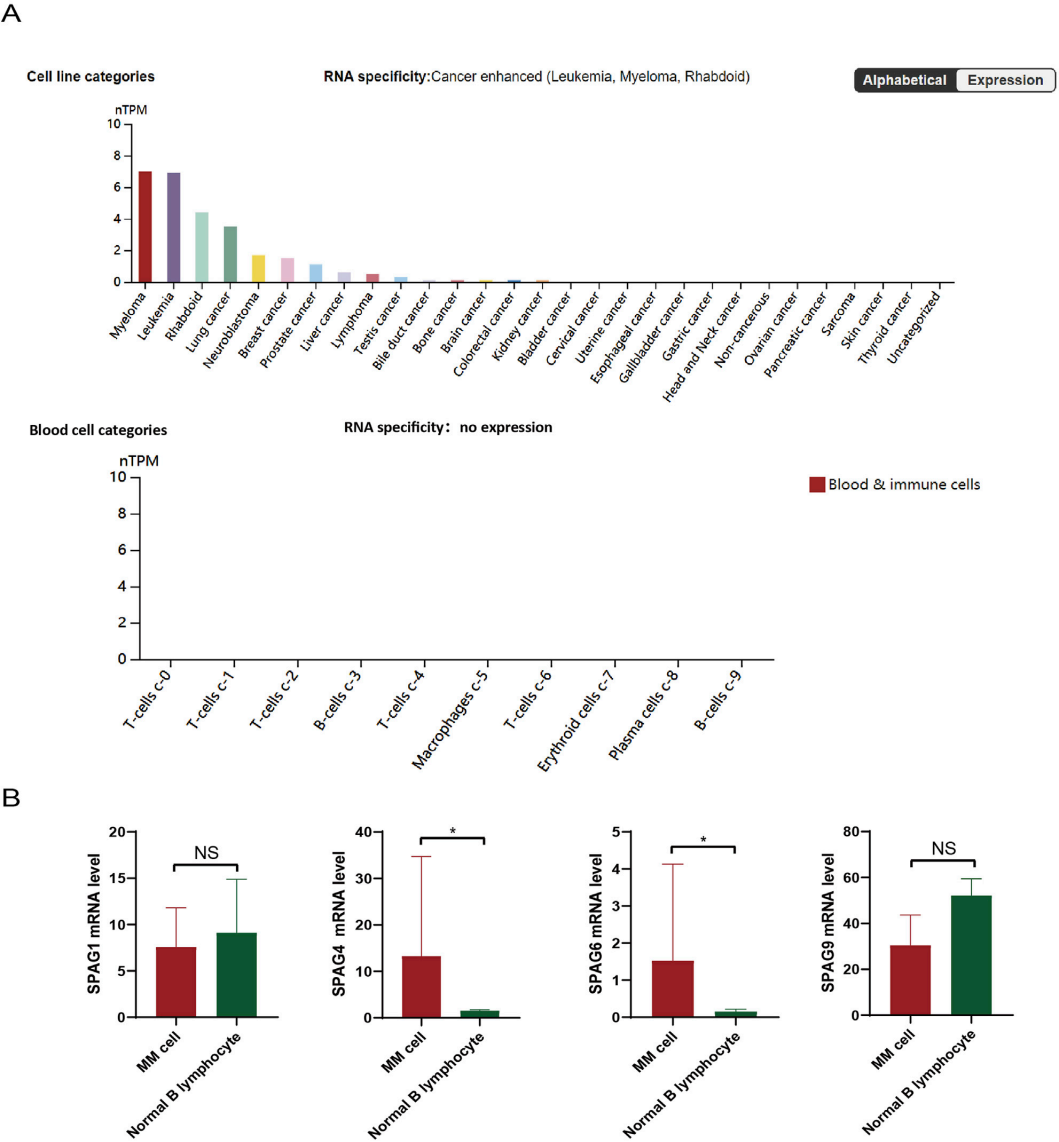
**(A)** Analysis of the Human Protein Atlas (HPA) database revealed the expression of SPAG6 in tumor cell lines, specifically, SPAG6 was found to be highly expressed in myeloma and leukemia cell lines. **(B)** SPAG6 expression within tumor cells and normal B cells, as documented in the HPA database.

In the Human Protein Atlas (HPA) database (https://www.proteinatlas.org), sequencing analysis revealed SPAG6 highly expressed in myeloma, leukemia, and rhabdoid tumor cell lines, especially in myeloma (Figure 1A). In contrast, there was either no expression or extremely low expression in normal blood cells and immune cells, including plasma cells (Figure 1A). However, whether SPAG6 functions as an oncogene or a tumor suppressor gene remains undetermined. Thus, this study aims to further explore SPAG6’s role in multiple myeloma and clarify the underlying mechanisms.

## 2 Material and methods

### 2.1 Patient samples

The patients enrolled in this study were from the First Affiliated Hospital of Chongqing Medical University. First of all, the diagnosis of pathological tissue biopsies consistent with plasmacytoma needs to be verified by two pathologists. The tissue sections from the pathological specimens were subjected to staining with hematoxylin-eosin and immunohistochemistry techniques. Subsequently, the expression of SPAG6 in the malignant tissues of plasmacytoma was to be examined. The newly diagnosed patients with multiple myeloma were selected to serve as the experimental cohort, with their diagnoses being confirmed based on the guidelines established by the International Myeloma Working Group (IMWG) (Sabattini et al., 2010). The staging standard used the International Staging System (ISS), Risk stratification adopts Mayo Stratification of Myeloma and Risk-Adapted Therapy (mSMART) version 3.0 (Gonsalves et al., 2019). Clinical characteristics including gender, age, ratio of monoclonal plasma cells in bone marrow, number of bone lesions, and chromosomal abnormalities were collected. The control cohort was patients who excluded hematological malignancy, solid tumors, and immune diseases. Bone marrow samples were taken from patients in the experimental group and the control group for the detection of SPAG6 expression levels. Bone marrow samples were collected from individuals in both the experimental cohort and control cohort to assess SPAG6 expression levels. Informed consent was obtained from all patients, and the study protocol was approved by the ethics committee of the patient’s hospital.

### 2.2 Pathological tissue staining technology

#### 2.2.1 Hematoxylin-eosin (HE) staining

The paraffin-embedded pathological tissues were cut into 4 μm thick slices in a paraffin microtome, unfolded with 30% alcohol, flattened in 40°C warm water, dried, and numbered. Submerge the sections in a dewaxing solution and subsequently in absolute ethanol to de-wax. The dewaxed paraffin sections were washed with water and stained with hematoxylin (cat. no. G1004; Wuhan Servicebio Technology Co., Ltd.), and then put into eosin stain (cat. no. G1004; Wuhan Servicebio Technology Co., Ltd.) for 6 min, then images were collected with a light microscope (Zeiss GmbH).

#### 2.2.2 Immunohistochemistry (IHC) staining

The paraffin sections were dewaxed, washed with water, and blocked with 3% bovine serum albumin (cat. no. G5001; Wuhan Service Technology Co., Ltd.) at room temperature for 30 min. Add primary antibody: Anti-SPAG6 (rabbit; dilution, 1:200; cat. no. Bs-12291R; BIOSS) and incubate overnight at 4°C. Then add a secondary antibody (goat anti-rabbit/goat anti-mouse; dilution, 1:200; cat.no. GB25303/GB21301; Wuhan Service Technology Co., Ltd.) to cover the tissue, and incubate at room temperature for 50 min. The staining outcomes were observed with the confocal laser scanning microscope (Eclipse Ti; Nikon Corporation). The digital pathology image analysis software Aipathwell was used to conduct the analysis. The immunoreactive score (IRS) was utilized to evaluate the immune response by employing tracing, color selection, and calculation.

### 2.3 Extraction of human bone marrow mononuclear cells (MNCs)

Human bone marrow fluid was collected and diluted with 2 mL PBS in EDTA anticoagulation tube. The fluid was added to a centrifuge tube with lymphocyte separation solution (Ficoll), and the MNCs layer was acquired following a 30-min low-speed centrifugation process. The lysate was added to the aforementioned centrifuge tubes, the contents were then thoroughly mixed and incubated on ice for 15 min. After the centrifugation and washing procedures, the MNCs were collected and stored in −80°C.

### 2.4 Cell lines and cell culture

The human multiple myeloma cell lines U266 and RPMI-8226, previously adopted in research, were utilized in this project (Li et al., 2020). All cells passed *mycoplasma* detection and short tandem repeats (STR) detection. The two cell lines were cultured in RPMI-1640 medium enriched with 10% fetal bovine serum (FBS), then transferred into culture flasks and placed in an incubator set at 37°C. The culture medium was refreshed after 24 h and subsequently 2–3 times, depending on the rate of cell proliferation.

### 2.5 Lentiviral vector construction

RNA interference (RNAi) technology was employed to disrupt the expression of SPAG6. Lentiviral vectors were procured from Gene Pharma.The lentiviral short - hairpin RNAs, shRNA 1 (targeting the sequence 5′ - GCC​ATA​AAG​AAT​ATC​CTG​CAA - 3′ of human SPAG6 mRNA) and shRNA2 (with the sequence 5′ - AAA​GCA​TTC​TCC​AGA​GTT​A - 3′), along with a negative control sequence (5′ - TTC​TCC​GAA​CGT​GTC​ACG​T - 3′), were cloned into a lentiviral vector to generate SPAG6 - knockdown constructs. In our study, shRNA 1 was used alone in certain experiments. To further enhance the stability of the knockdown efficiency, a combination of shRNA 1 and shRNA 2 was utilized in the mechanistic research of U266 cells for validation. Additionally, the full - length coding sequence of SPAG6 (NM - 012443.4) was cloned into a lentiviral expression vector to create the SPAG6 - overexpression construct.

### 2.6 The messenger RNA (mRNA) expression level

The expression level of SPAG6 was precisely quantified via real - time quantitative polymerase chain reaction (RT - qPCR). Cryopreserved cells underwent rapid thawing in a 40°C water bath, followed by centrifugation. Subsequently, the cells were meticulously washed, after that, an RNA extraction reagent was introduced, along with 0.2 mL of chloroform. Following centrifugation at 4°C, the RNA was carefully collected. Next, the isolated RNA was reverse - transcribed into complementary DNA (cDNA) in strict accordance with a standardized protocol. Real - time qPCR was then carried out under optimized experimental conditions. The relative expression levels of SPAG6 and the internal reference gene were calculated using the 2^(−ΔΔCt) method.

### 2.7 Cell counting kit (CCK - 8) assay and the 5 - ethynyl - 2′ - deoxyuridine (EdU) staining

To accurately evaluate cell proliferation, CCK - 8 assay was used. 100 μL cell suspensions, free of bubbles, were seeded into each well of 96 - well plates. The plates were incubated at 37°C in 5% CO_2_ for 0, 12, 24, and 48 h. After incubation, 10 μL CCK - 8 solution was added and gently mixed. The absorbance at 450 nm was measured by a microplate reader to calculate cell viability.

Cell proliferation was further verified by EdU staining. Cells were seeded in six - well plates and incubated overnight. Equal - volume EdU working solution was added, followed by a 2 hour incubation. After centrifugation, cell suspensions were made into slides and fixed with 4% paraformaldehyde. Nuclear staining was done with Hoechst - 33342. Finally, slides were mounted with anti - quenching agent. Laser confocal microscopy detected EdU - positive cells. EdU appears red in U266 cells but green in RPMI-8226 cells according to the different staining reagent.

### 2.8 Cell apoptosis

Cell apoptosis was detected following this protocol. First, cells were washed with PBS and centrifuged. Then, 7 - AAD staining solution was added, and the samples were incubated in the dark for 5 min. Next, 450 μL of Binding Buffer was added and mixed, followed by AnnexinV - PE addition. The mixture was gently pipetted and incubated for 15 min. Finally, a flow cytometer analyzed the results.

Additionally, RPMI - 8226 and SPAG6 - stably transfected cells were collected. A 50 μmol/L apoptosis inducer (2 - ME2) was added, and cells were incubated for 2 h. Subsequent steps adhered to the apoptosis detection kit protocol, followed by flow cytometry.

### 2.9 Transwell assay

When cells in each group entered the log - phase, they were transferred to centrifuge tubes, centrifuged, and the supernatant discarded. Next, the washed cells were resuspended in serum - free medium at a density of 1 × 10^5^ cells/mL. For a 24 - well Transwell, 500 μL of medium containing 15% fetal bovine serum was added to the lower chamber, and 200 μL of the cell suspension (2 × 10^4^ cells/well) to the upper chamber. After 24 hour incubation, the Transwell was processed as follows: discard the medium, wash with PBS, fix, stain with 0.1% crystal violet, and finally, count the migrated cells under a microscope.

### 2.10 RNA sequencing (RNA - seq)

U266 samples were classified into CON (negative control), V (Sh - negative control interference), and SI (Sh - SPAG6 virus interference) groups. Pairwise comparisons (CON vs. V, CON vs. SI, V vs. SI) were performed for transcriptome profiling. Cells were centrifuged, washed, and lysed with 1 mL Trizol. RNA was extracted from lysates and denatured for cDNA generation. The cDNA was end - repaired, A - tailed, adapter - ligated, and PCR - amplified. The library was quality - assessed, circularized, and DNA nanospheres (DNBs) sequenced via Combinatorial Probe - Anchor Synthesis (cPAS).

### 2.11 Analysis of RNA - seq data

Raw data was filtered by SOAPnuke (v1.5.6) and analyzed using the Dr.Tom system. It was aligned by HISAT2 (v2.1.0) and Bowtie2 (v2.3.4.3), and gene expression was quantified by RSEM (v1.3.1). DESeq2 (v1.4.5) detected differential genes, and Phyper was used for GO/KEGG enrichment. Specifically, the Phyper function (based on the hypergeometric test) was used for enrichment analyses of GO (http://www.geneontology.org/) and KEGG (https://www.kegg.jp/) on differentially expressed genes.

### 2.12 Co - Immunoprecipitation assay (Co - IP)

The Co - IP followed the Protein A/G Immunoprecipitation Kit (cat. no. M2400; Solarbio Science and Technology Co., Ltd.). First, prepare working solutions: mix lysis buffer with protease inhibitor (100:1) and dilute TBS (1:9). For cell samples, centrifuge the cell suspension at 1,000 rpm for 5 min, wash the cells once. Then resuspend the cells and centrifuge at 3,000 rpm for 3 min. Add 100 μL of lysis buffer containing protease inhibitor, lyse on ice, and then centrifuge and collect the supernatant. To prepare magnetic beads, resuspend them, add to the sample (1:25). For antibody - bead binding, make a 25 μg/mL antibody solution, add 200 μL to magnetic bead suspension, incubate at room temperature for 1 h. Then place the tube on the magnetic rack and separate it magnetically for 10 s, For immunoprecipitation, mix the protein sample with the bead - antibody complex (25:1), incubate at 25°C. Wash with lysis buffer. Elute with SDS - PAGE loading buffer (1:5) at 95°C, and collect the supernatant for further analysis.

### 2.13 Western blot (WB) protocol

#### 2.13.1 Reagent preparation

Prepare PBS (pH 7.2, autoclaved at 121°C), 10 mM PMSF (stored at −20°C), 10% SDS, 10% APS (4°C), Tris buffers (pH 8.8/6.8, 4°C), 1× Tris-glycine electrophoresis buffer, 1× TBST (0.1% Tween-20), transfer buffer (20% methanol), and 5% non-fat milk in TBST for blocking. Gel concentrations are selected by target protein size.

#### 2.13.2 Total protein extraction

Cells were lysed in ice-cold RIPA buffer (50 mM Tris-HCl, pH 7.4, 150 mM NaCl, 1% NP-40, 0.5% sodium deoxycholate, 0.1% SDS) supplemented with 1% PMSF. After 30 min of lysis on ice with intermittent vortexing every 5 min, lysates were centrifuged for 15 min at 4°C. The supernatant was collected, and protein concentration was quantified using the BCA assay (Thermo Fisher Scientific). Proteins were denatured by boiling with 1× SDS loading buffer (sample:buffer = 4:1) for 10 min and stored at −20°C.

#### 2.13.3 WB procedure

Assemble gel plates, pour separating gel (sealed with ethanol), and polymerize. Add stacking gel, insert comb, and load 40 μg protein per well. Electrophorese at 80 V until bromophenol blue enters stacking gel, then 120 V until dye nears the bottom. Transfer proteins to activated PVDF membrane at 250 mA, 4°C (1–3 h, adjusted by protein size). Block membrane with 5% milk in TBST for 1 h, incubate with primary antibodies (1:1,000 dilution, rabbit, cat. no. Bs-12291R; BIOSS) at 4°C overnight, and wash 4× with TBST (5 min each). Incubate with HRP-conjugated secondary antibodies (1:1,000) for 1 h at RT, wash similarly, and detect using ECL reagent (A:B = 1:1) via chemiluminescence imaging.

### 2.14 Statistical analysis

Statistical analysis was completed with SPSS 26.0 and GraphPad Prism (Version 8.0) software. The measurement units are described as median (range) or mean ± standard error ‾x ±s), the difference between two different groups of measurement data is analyzed using a T-test or non-parametric test, ANOVA analysis of variance is used to compare multiple groups of measurement data. Post hoc testing is conducted utilizing the Bonferroni test. Receiver operating characteristic (ROC) curve analysis is used to evaluate the discriminating ability between the experimental and the control group. Prognostic analysis was performed using the Kaplan-Meier survival curve and Log-rank test, the multivariate survival analysis was conducted employing COX proportional hazard model regression analysis. *P* < 0.05 suggests that the difference is statistically significant. When the *p* - value ranges from 0.04 to 0.05, Bayesian statistics can offer extra insights. A Bayes Factor >1 implies stronger support for the alternative hypothesis, suggesting statistical significance.

## 3 Results

### 3.1 SPAG6 is overexpressed in MM cell lines

Statistical analysis of tumor cell line sequencing samples from the Human Protein Atlas (HPA) database (https://www.proteinatlas.org) showed the expression of SPAG 4 and SPAG6 in the SPAG family was significantly different between myeloma cell lines and normal B cell lines (*P* < 0.05) (Figure 1B). Although SPAG 4 is a potential biomarker for a variety of solid tumors (Kroemer and Pouyssegur, 2008; Yin et al., 2021), many studies have shown that SPAG6 is more associated with hematological tumors (Jiang et al., 2019; Zhang M. et al., 2020). Therefore, this study will further explore the possible role of SPAG6 in patients with MM.

### 3.2 SPAG6 is expressed in pathological tissues of patients with newly diagnosed MM

**FIGURE 2:**
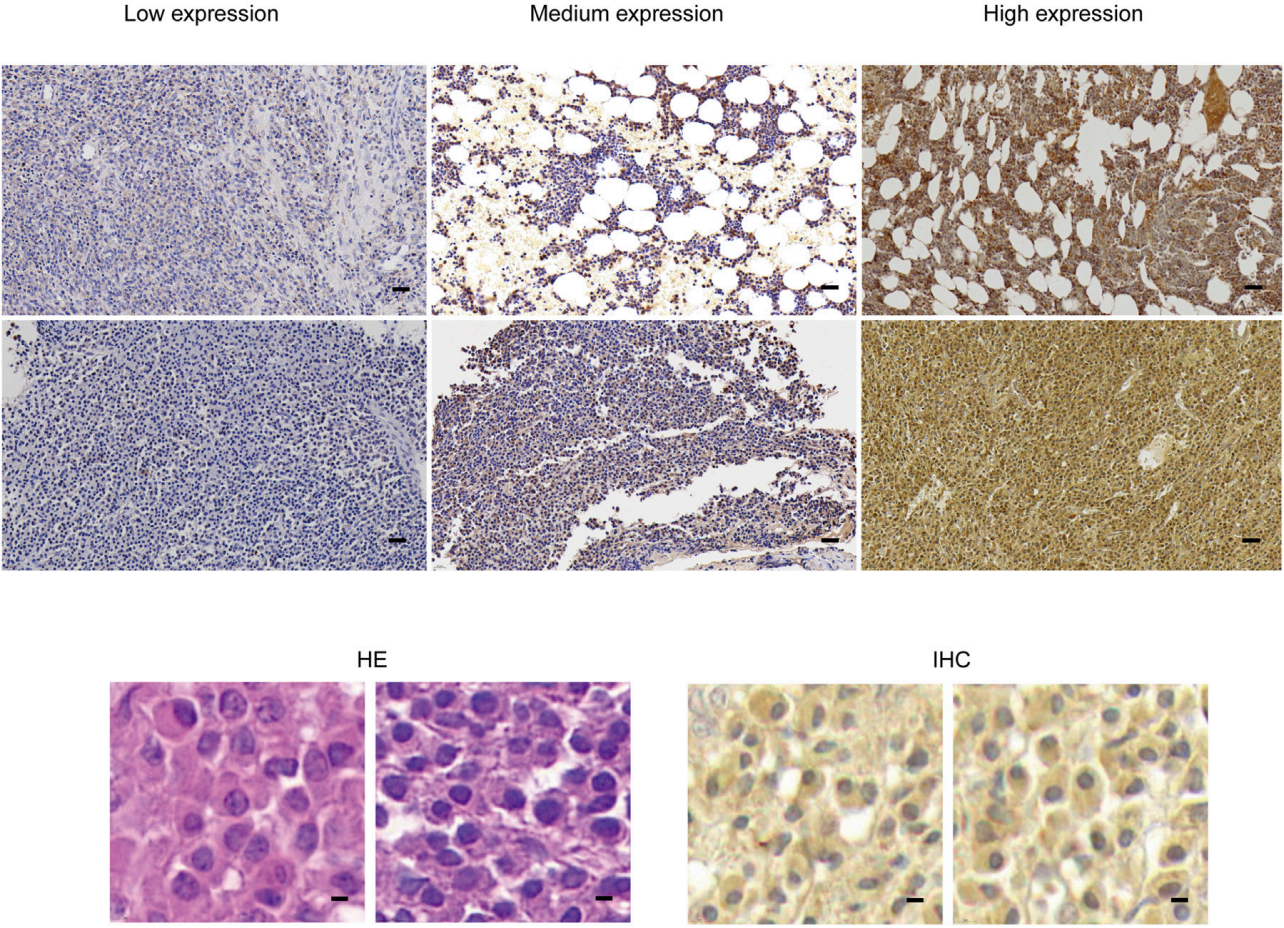
In pathological specimens obtained from patients with plasmacytoma, HE staining and immunohistochemical staining were performed, dark brown color of immunohistochemical staining indicates positive SPAG6 expression. Under lower magnification (scale bar: 20 µm), higher magnification (scale bar: 5μm) demonstrated the positive expression of SPAG6. HE, Hematoxylin and Eosin.

This section of the study compiled data from 22 patients with pathologically confirmed plasmacytoma diagnoses. Among them, two patients were ultimately diagnosed with primary extraosseous/extramedullary plasmacytoma (EMP), 12 were diagnosed with multiple myeloma (MM), and 8 were diagnosed with solitary bone plasmacytoma (SBP). The basic clinical data of patients were presented in Table 1. The pathological tissue sections underwent HE staining as well as immunohistochemical staining for SPAG6. The representative immunohistochemical staining results are displayed in Figure 2. Under high magnification, the positive expression of SPAG6 was predominantly observed within the cytoplasm of cells (Figure 2). Patients with EMP were not included in the statistical analysis due to a limited sample size. Through the statistical analysis, the median positive cell ratio in patients with MM was 54.65% (7.45%–94.24%), and in patients with SBP was 14.76% (4.22%–70.56%), the two groups exhibited a significant difference (*P* = 0.04). Upon the final IRS assessment, the median IRS score for SPAG6 in patients with MM was five points (2–8), whereas in patients with SBP, it was two points (0–6), Notably, there was a significant difference between the two groups (*P* = 0.02) (Table 1). The findings suggested that if SPAG6 influences the function of plasma cell tumors, this molecule might play a role in promoting their development.

**TABLE 1:** Major clinical characteristics of patients with plasmoma.

Variable	EMP	MM	SBP	*P* (MM and SBP)
Number (%)	2 (9.10%)	12 (54.55%)	8 (36.36%)	
Sex (male/female)	2	7/5	5/3	0.28
Median age (rang)	50.5 (45–56)	61.5 (42–72)	53 (45–72)	0.12
Tissue site	Spleen, subdural tissue	Skeletal tissue	Skeletal tissue	
IHC (SPAG6)				
Median positive cells, % (rang)	13.57% (4.05%–23.1%)	54.65% (7.45%–94.24%)	14.76% (4.22%–70.56%)	0.04*
IRS score	1 (0–2)	5 (2–8)	2 (0–6)	0.02*

*Statistical difference (*p* < 0.05) was observed.

### 3.3 Basic information about patients

**FIGURE 3:**
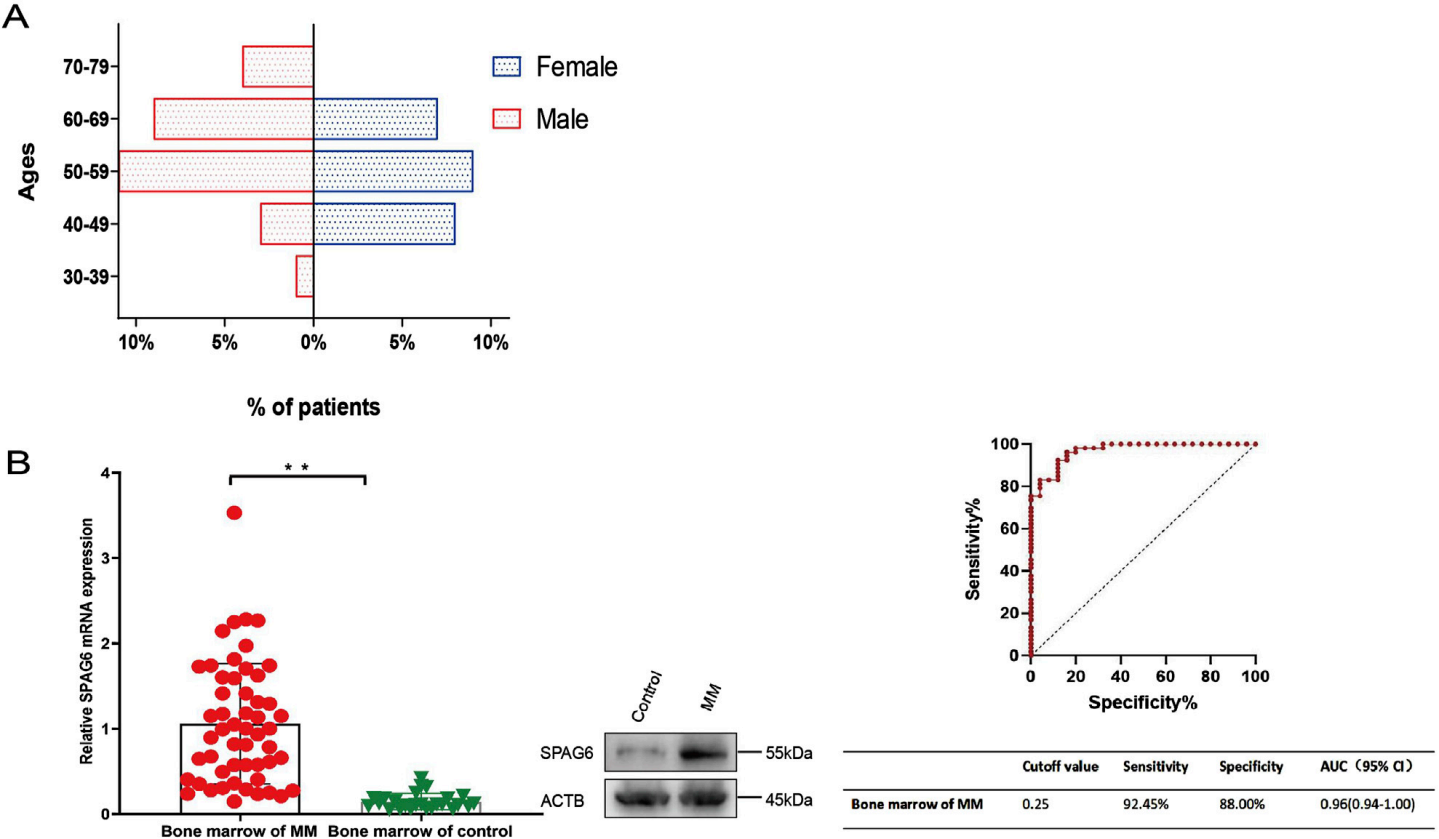
**(A)** Age structure diagram of patients with multiple myeloma. **(B)** SPAG6 expression in the bone marrow of multiple myeloma patients was significantly higher than that in the control group. Receiver Operating Characteristic (ROC) curves were constructed to determine the optimal cut-off value for the relative expression levels of SPAG6 mRNA in bone marrow, which effectively distinguished the multiple myeloma group from the control group. *p < 0.05, **p < 0.01.

A total of 53 patients diagnosed with multiple myeloma (MM) were enrolled in this phase of the study, 56.60% (30/53) of the participants were male, 43.40% (23/53) were female, the median age was 56 years (38–76 years). The age distribution chart indicated that individuals aged 50 to 59 were the most vulnerable demographic among patients diagnosed with multiple myeloma, accounting for 39.62% (21/53), followed by was 60–69 year-old group, accounting for 30.19% (16/53). The female patients were predominantly in the 40–69 age range, whereas the age distribution of male patients was broader, with the disease affecting individuals both under 40 and over 70 years of age (Figure 3A). The detailed basic information of the enrolled patients is shown in Table 2. The control group consisted of 25 patients, and flow cytometry immunophenotyping revealed no phenotypically abnormal plasma cells.

**TABLE 2:** Clinical characteristics of 53 patients with MM.

Clinical variables	Median (range or count%)
Total number	53
Gender (female/male)	23/30
Median age (years)	56 (38–76)
Median bone marrow plasma cells (%)	19 (1.13–70)
Symptoms	
Hypercalcemia	9 (16.98%)
Bony destruction	39 (73.58%)
Renal function abnormal	14 (26.42%)
Anemia	7 (13.21%)
Extramedullary infiltration	3.77% (2/53)
M-protein	
IgG	26 (49.06%)
IgA	13 (24.53%)
IgM	1 (1.89%)
Light chain	13 (24.53%)
Abnormal chromosome site (number)	27 (50.94%)
1	15 (55.56%)
2	8 (29.63%)
≥3	4 (14.81%)
Risk stratification	
High risk	29 (54.72%)
Standard risk	24 (45.28%)
Treatments	
Stem cell transplantation	15 (28.30%)
Chemotherapy	38 (71.70%)
Median follow-up term (months)	1 (8–60)

^a^ MM, multiple myeloma M-protein, monoclonal protein.

### 3.4 Expression levels of SPAG6 in patients with MM

The average relative expression level of SPAG6 mRNA in the bone marrow of 53 patients with MM was 1.06 ± 0.71, whereas in the control group, it was 0.15 ± 0.10. Further analysis showed there was a significant difference between the two groups (*P* < 0.01). The expression level of SPAG6 protein in the bone marrow of multiple myeloma (MM) patients was observed to be elevated compared to that of the control group (Figure 3B). The results of ROC curve analysis showed the cutoff value of SPAG6 expression to distinguish MM patients from controls was 0.25, the sensitivity was 92.45%, the specificity was 88%, and the area under the curve (AUC) was 0.97 (0.94–1.00) (Figure 3B).

### 3.5 SPAG6 expression and clinical characteristics in patients with MM

#### 3.5.1 The correlation between SPAG6 expression and clinical characteristics of patients with MM

**FIGURE 4:**
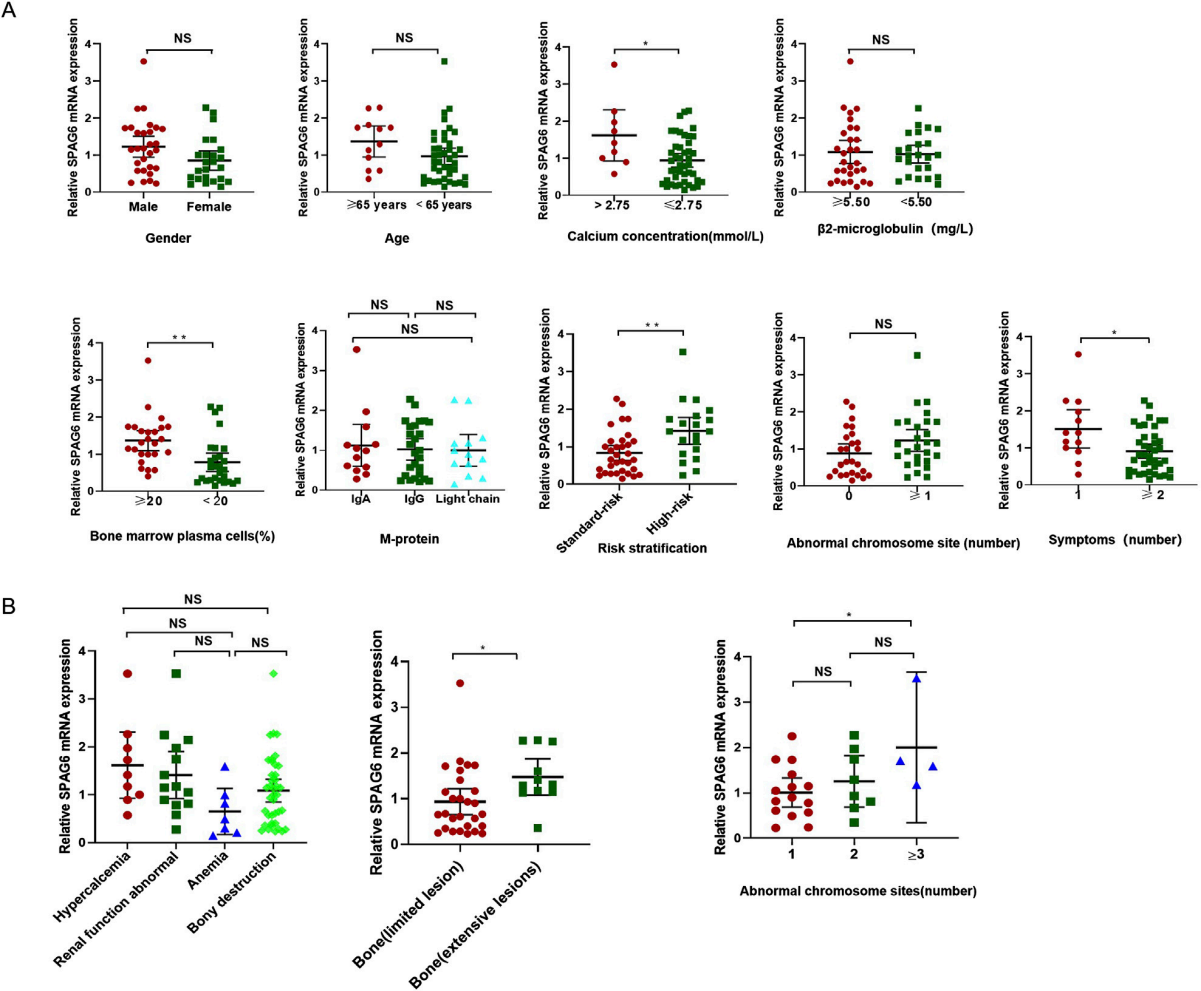
**(A)** SPAG6 mRNA expression was higher in patients with serum calcium >2.75 mmol/L, bone marrow plasma cell >20%, high-risk multiple myeloma, and ≥2 organ-related symptoms. **(B)** Correlation of SPAG6 mRNA relative expression levels with organ-related symptoms, skeletal lesion distribution, and chromosomal abnormalities in multiple myeloma patients.

To comprehend the function of SPAG6 in multiple myeloma (MM) patients, we examined the correlation between the relative expression levels of SPAG6 mRNA and the clinical features of MM patients. The analysis revealed no correlation between the relative expression of SPAG6 mRNA and the age, gender, and β2 microglobulin levels of MM patients (*P* ≥ 0.05) (Table 3). However, in patients with blood calcium concentration exceeding 2.75 mmol/L, bone marrow plasma cell ratio greater than 20%, high-risk MM, and more than two symptoms of target organ damage, the relative expression of SPAG6 mRNA was found to be elevated, with statistically significant differences (*P* < 0.05) (Figure 4A).

**TABLE 3:** Relationship between relative SPAG6 mRNA expression and clinical characteristics of MM patients.

Clinical variables	Parameter or number	No. (%)	Mean of SPAG6 expression	*P* value
Gender	Male	30 (56.60%)	1.23 ± 0.74	0.05
	Female	23 (43.40%)	0.85 ± 0.62	
Age (years)	≥65	12 (22.64%)	1.37 0.66	0.08
	<65	41 (77.36%)	0.97 0.70	
Calcium concentration (mmol/L)	>2.75	9 (16.98%)	1.62 ± 0.90	0.01*
	≤2.75	44 (83.02%)	0.94 ± 0.61	
β2-microglobulin (mg/L)	≥5.5	29 (54.72%)	1.02 ± 0.56	0.77
	<5.5	24 (45.28%)	1.08 ± 0.82	
Bone marrow plasma cells (%)	≥20	25 (47.17%)	1.37 ± 0.65	0.00**
	<20	28 (52.83%)	0.78 ± 0.64	
M-protein	IgA	13 (24.53%)	1.12 ± 0.87	0.89
	IgG	26 (49.06%)	1.02 ± 0.66	
	Light chain	13 (24.53%)	1.00 ± 0.66	
Risk stratification	High risk	20 (54.72%)	1.43 ± 0.77	0.00**
	Standard risk	33 (45.28%)	0.83 ± 0.57	
Abnormal chromosome site	0	26 (49.06%)	0.88 ± 0.63	0.07
	≥1	27 (50.94%)	1.23 ± 0.74	
Symptoms	1	40 (75.47%)	0.91 ± 0.59	0.01*
	≥2	13 (24.53%)	1.51 ± 0.85	

*Statistical difference (*p* < 0.05) was observed, **Statistical difference (*p* < 0.01) was observed.

#### 3.5.2 The correlation between SPAG6 expression and symptomatology

The extent and location of organ damage in patients with MM can vary, leading to a range of symptoms. Some individuals experience localized bone infiltration, whereas others suffer from extensive skeletal destruction (≥3). Therefore, in the study, the four major symptoms caused by target organ damage were classified in detail and the expression of SPAG6 was analyzed. The results showed that there was no significant difference in the average expression of SPAG6 between the four common symptoms (*P* = 0.05), but among bone lesions, the average relative expression of SPAG6 was higher in patients with extensive bone lesions than local lesions (*P* = 0.04) (Table 4; Figure 4B).

**TABLE 4:** Relationship between relative expression of SPAG6 mRNA and different symptoms in patients with MM.

Symptoms	Mean of *SPAG6* expression	*F* value	*P* value
Hypercalcemia	1.62 ± 0.90	2.74	0.05
Renal function abnormal	1.41 ± 0.85		
Anemia	0.65 ± 0.52		
Bony destruction	1.09 ± 0.73		
Bone (limited lesion)	0.93 ± 0.73		0.04*
Bone (extensive lesions)	1.48 ± 0.59		

*Statistical difference (*p* < 0.05) was observed.

#### 3.5.3 The correlation between SPAG6 expression and cytogenetics

We also performed further analyses on the number of high-risk cytogenetic abnormalities. The research results showed that the relative expression level of SPAG6 was also different depending on the number of chromosomal abnormality sites. The relative expression level of SPAG6 mRNA of patients with three abnormal sites is higher than that of patients with only 1 abnormal site (*P* = 0.048) (Table 5) (Figure 4B).

**TABLE 5:** Relationship between SPAG6 mRNA relative expression levels and cytogenetic abnormalities in MM patients.

Abnormal chromosome sites (number)	Mean of SPAG6 expression	*F* value	*P* value
1	1.01 ± 0.58	3.36	0.05
2	1.26 ± 0.68		
≥3	2.00 ± 1.04		
1 vs. ≥3			0.048*

Vs. versus, *Statistical difference (*p* < 0.05) was observed.

### 3.6 Prognostic analysis of patients with MM

**FIGURE 5:**
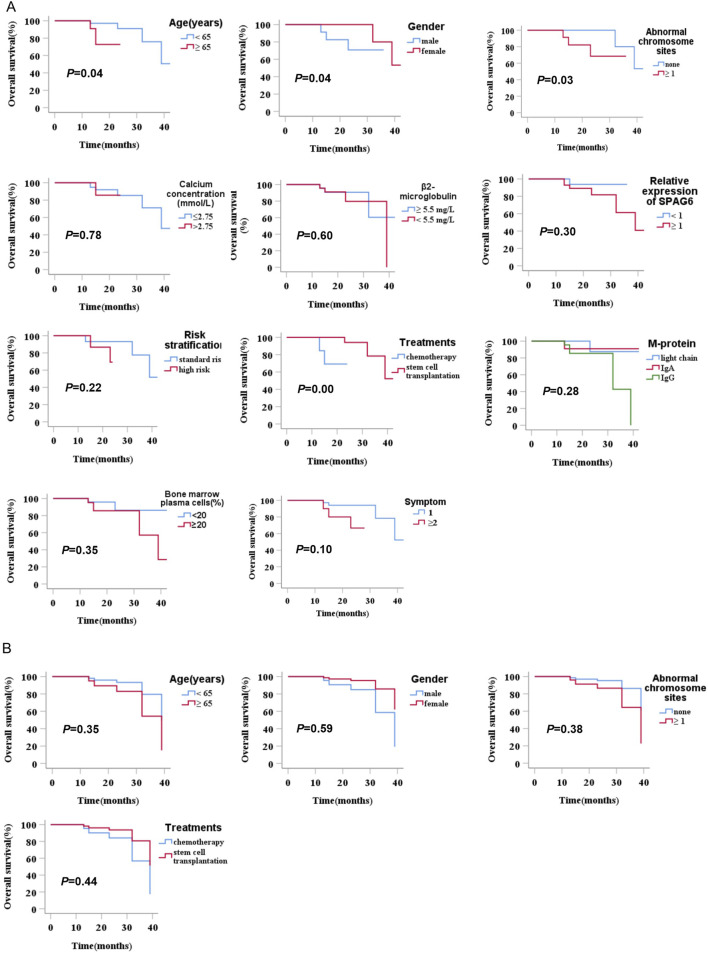
**(A)** Univariate analysis of the prognostic impact of relative SPAG6 mRNA expression levels and their association with clinical characteristics in multiple myeloma patients. **(B)** Multivariate analysis of the prognostic impact of relative SPAG6 mRNA expression levels and their association with clinical characteristics in multiple myeloma patients.

In this research, we explored the connection between the clinical manifestations and prognostic outcomes of all individuals with MM. The results of the univariate analysis indicated a significant difference in survival time among patients with different ages, gender, chromosomal abnormalities, and treatment methods (*P* < 0.05). The survival time of patients aged ≥65 years was shorter than that of patients aged <65 years (*P* = 0.04). The survival time of male patients was shorter than that of female patients (*P* = 0.04). Patients with cytogenetic abnormalities (number of chromosomal abnormality sites ≥1) had shorter survival times than patients without cytogenetic abnormalities (*P* = 0.03). In treatment methods, patients who underwent hematopoietic stem cell transplantation had a better survival time than those who received conventional chemotherapy (*P* = 0.00), while the relative expression of SPAG6 (≥1 versus <1) did not significantly affect survival time (*P* = 0.30) (Table 6) (Figure 5A). Upon conducting a multivariate analysis, it was observed that the parameters that exhibited significant differences in the univariate analysis did not notably influence survival time (*P* ≥ 0.05) (Table 6) (Figure 5B).

**TABLE 6:** The effect of clinical characteristics and the relative expression level of SPAG6 mRNA on the prognosis of MM patients.

Variables	Survival analysis		
	Univariate analysis *P* value	Multivariate analysis HR (CI 95%)	*P* value
Age (years) (≥65 versus <65)	0.04*	0.399 (0.059–2.718)	0.348
Gender (male versus female)	0.04*	2.098 (0.145–30.303)	0.587
Chromosome abnormal sites (none versus ≥1)	0.03*	0.381 (0.029–3.891)	0.381
Calcium concentration (mmol/L) (>2.75 versus ≤2.75)	0.78		
β2-microglobulin (mg/L) (≥5.50 versus <5.50)	0.60		
Relative SPAG6 mRNA expression level (≥1 versus <1)	0.30		
Risk stratification (high-risk versus standard risk)	0.22		
M-protein types	0.28		
Symptom (1 versus ≥2)	0.10		
Bone marrow plasma cells (%) (≥20 versus <20)	0.35		
Treatments (stem cell transplants versus chemotherapy)	0.00**	2.237 (0.285–17.565)	0.444

*Statistical difference (*p* < 0.05) was observed, **Statistical difference (*p* < 0.01) was observed COX, proportional hazard regression analysis was used for multivariate analysis.

### 3.7 SPAG6 promotes the proliferation and migration of MM cells

**FIGURE 6:**
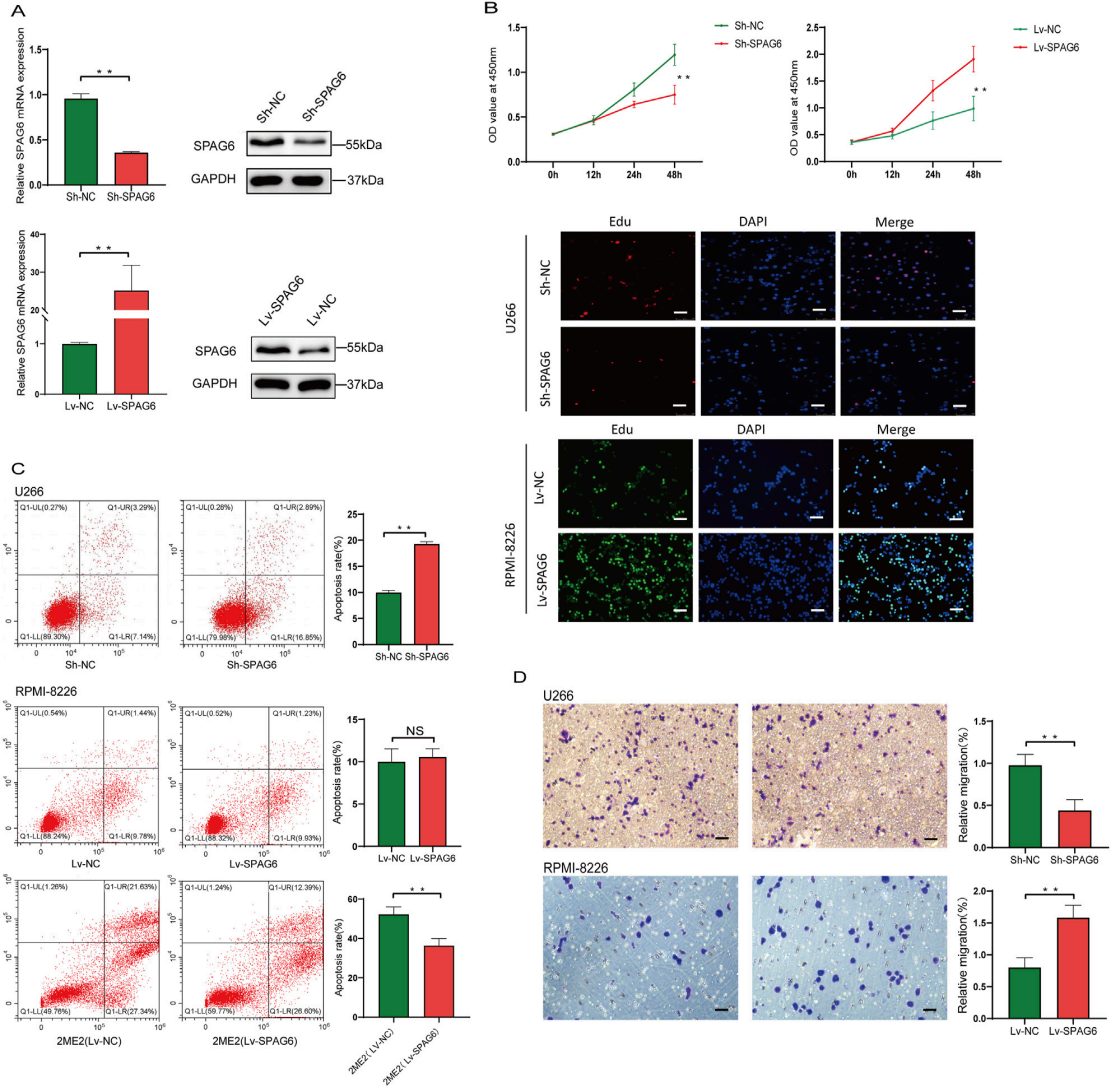
**(A)** Establishment of SPAG6 overexpression and knockdown in U266 and RPMI-8226 cell lines. **(B)** Proliferation of U266 and RPMI-8226 cells was evaluated by CCK-8 and EdU assays (scale bar 25 µm). **(C)** Apoptosis of U266 cells was analyzed by flow cytometry. **(D)** MM cells migration was determined by Transwell assay. (scale bar 25 µm).

Based on the expression levels of SPAG6 in U266 and RPMI-8226 cells, we successfully generated stable U266 cell lines with SPAG6 knockdown (designated as Sh-SPAG6) and RPMI-8226 cell lines with SPAG6 overexpression (designated as Lv-SPAG6). The experiments were repeated at least three times with similar results, and the representative images were shown in Figure 6A. The CCK-8 assay confirmed that the suppression of SPAG6 expression resulted in inhibited cell proliferation in the U266 cell line, while cell proliferation ability increased in RPMI-8226 with upregulated SPAG6, and EdU assay verified similar results (Figure 6B).

Furthermore, flow cytometry analysis using AnnexinV-PE and 7-AAD staining revealed that downregulated SPAG6 in U266 cells led to an increase in the proportion of apoptotic cells, particularly the late apoptotic cells. In RPMI-8226 cells with upregulated SPAG6, the apoptosis was similar to the control cells (*P* ≥ 0.05). However, the addition of the apoptosis-inducing agent 2-ME2 promoted the apoptosis of the control cells, whereas the RPMI-8226 cells with upregulated SPAG6 did not display a significant response, the late apoptotic cells were less than the control group (*P* < 0.01), which indicated RPMI-8226 cells increased the resistance to apoptotic inducer following forced expression of SPAG6 (Figure 6C).

In the initial phase of our study, we observed that the expression of SPAG6 was elevated in patients exhibiting extensive bone invasion. Subsequently, we conducted a Transwell migration assay to assess the migratory capacity of the cells. Consistent with expectation, the cell migration was a remarkable increase in RPMI-8226 cells with upregulation of SPAG6, while knockdown of SPAG6 seemed to impair the migratory ability of U266 cells (Figure 6D).

### 3.8 Mechanism of SPAG6 in MM cells

**FIGURE 7:**
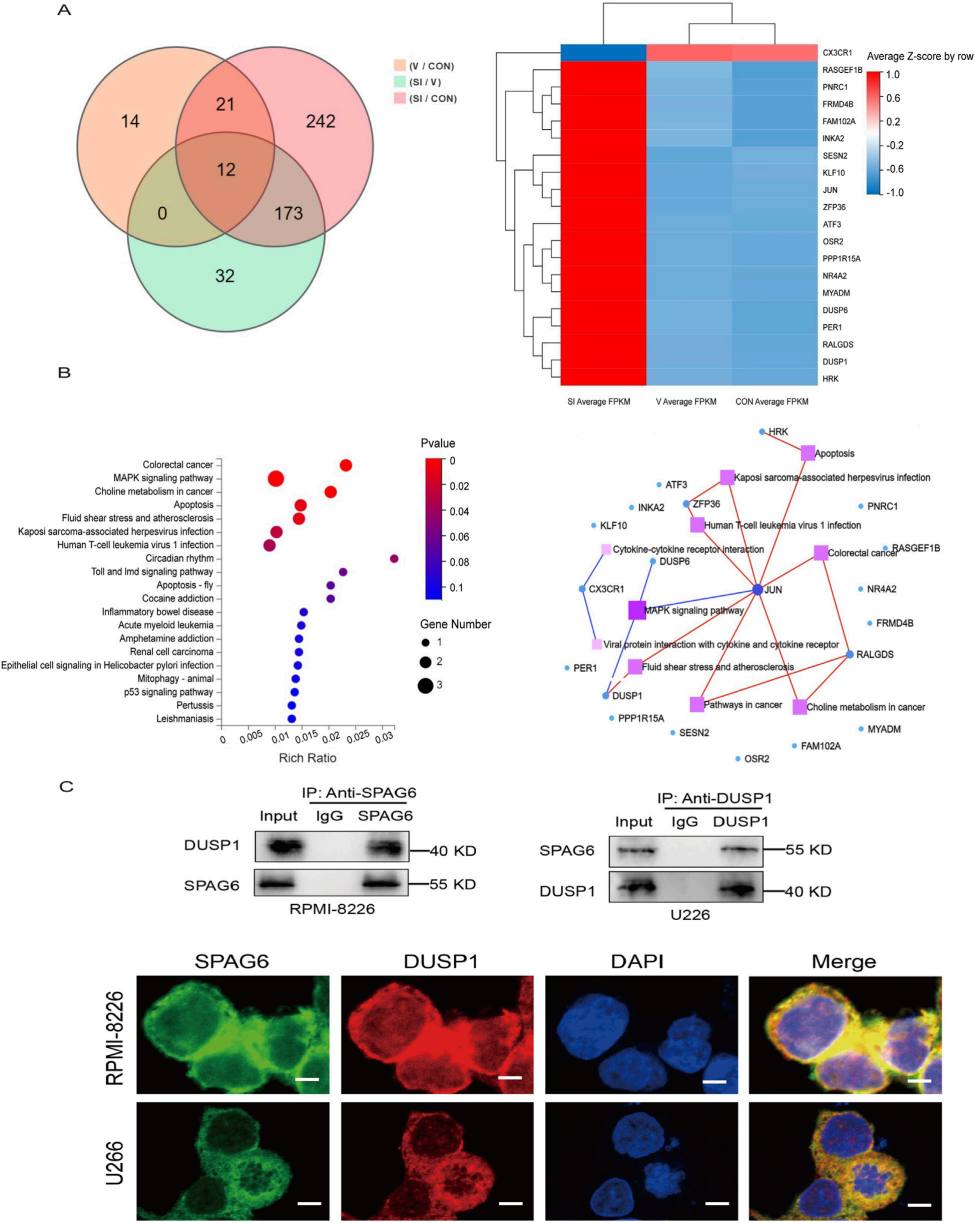
**(A)** Overview of transcriptome sequencing of MM cells, Wayn diagram and gene expression cluster heat map showed the differential genes. **(B)** KEGG pathway enrichment map and KEGG pathway network map. **(C)** The interaction between SPAG6 and DUSP1 was confirmed by Co-IP in the cell lines and cell images were displayed by a posterior laser confocal microscopy after immunofluorescence staining (scale bar 5 µm). Fluorescence imaging of SPAG6 (green), DUSP1 (red), and DAPI (blue) in the cells, green and red fluorescence were fused to yellow fluorescence, and red and blue were fused to purple fluorescence.

To further explore the mechanism of SPAG6 in MM, we performed mRNA sequencing in different states of U266 cells. The U266 cells were divided into blank control group (CON), control virus-transfection group (V), and knockdown SPAG6 group (SI), each group was set up with three biological replicates for mRNA sequencing. 173 differential genes were screened out (Figure 7A), and the top 20 genes with the largest difference between V and SI groups but no significant difference between CON and V were selected for functional and pathway analysis (Figure 7B). The expression and *P* value of the top 10 differential expression genes in each group are shown in Table 7.

**TABLE 7:** Expression status and *P*-value of the top 10 genes with expression differences in each group.

Gene symbol	P (SI/CON)	P (V/CON)	P (SI/V)	SI average	V average	CON average
PPP1R15A	4.45E-168	0.39	1.23E-142	35.93	3.88	3.44
MYADM	7.80E-133	0.68	2.58E-115	34.18	6.03	5.7
ATF3	5.93E-115	0.89	1.20E-116	26.57	3.79	3.71
DUSP1	1.82E-91	0.32	4.58E-77	28.82	4.22	3.67
ZFP36	2.71E-70	0.49	2.14E-78	32.82	8.55	8.87
FAM102A	1.62E-61	0.18	8.30E-59	21.31	8.71	7.67
JUN	1.06E-60	0.20	1.17E-61	36.36	7.75	8.24
CX3CR1	1.78E-50	0.77	2.07E-46	108.05	225.39	222.09
KLF10	4.18E-42	0.37	3.29E-36	36.7	16.33	16.78
DUSP6	2.61E-40	0.46	3.02E-37	13.59	4.90	4.52

Based on the literature, the MAPK pathway is one of the most widely regulated pathways in human tumor signaling and plays a vital role in controlling cell proliferation, differentiation, and tumor progression (Uprety and Adjei, 2020). Research has shown the DUSP1 gene is associated with the proliferation, apoptosis, development, and drug resistance of viral tumors (Zandi et al., 2022; Martínez-Martínez et al., 2021). Upon conducting KEGG pathway analysis, the gene DUSP1 was selected for further investigation. To verify the interaction between SPAG6 and DUSP1, Different antibodies were selected for immunoprecipitation experiment in U266 and RPMI-8226 cells. As expected, the interaction between SPAG6 and DUSP1 was detected (Figure 7C). Meanwhile, laser confocal microscopy confirmed intracellular proteins SPAG6 and DUSP1 were co-localized in the cytoplasm and the cell membrane, with DUSP1 also expressed in the nucleus (Figure 7C).

### 3.9 SPAG6 activates the downstream MAPK/ERK signaling pathway through DUSP1

**FIGURE 8:**
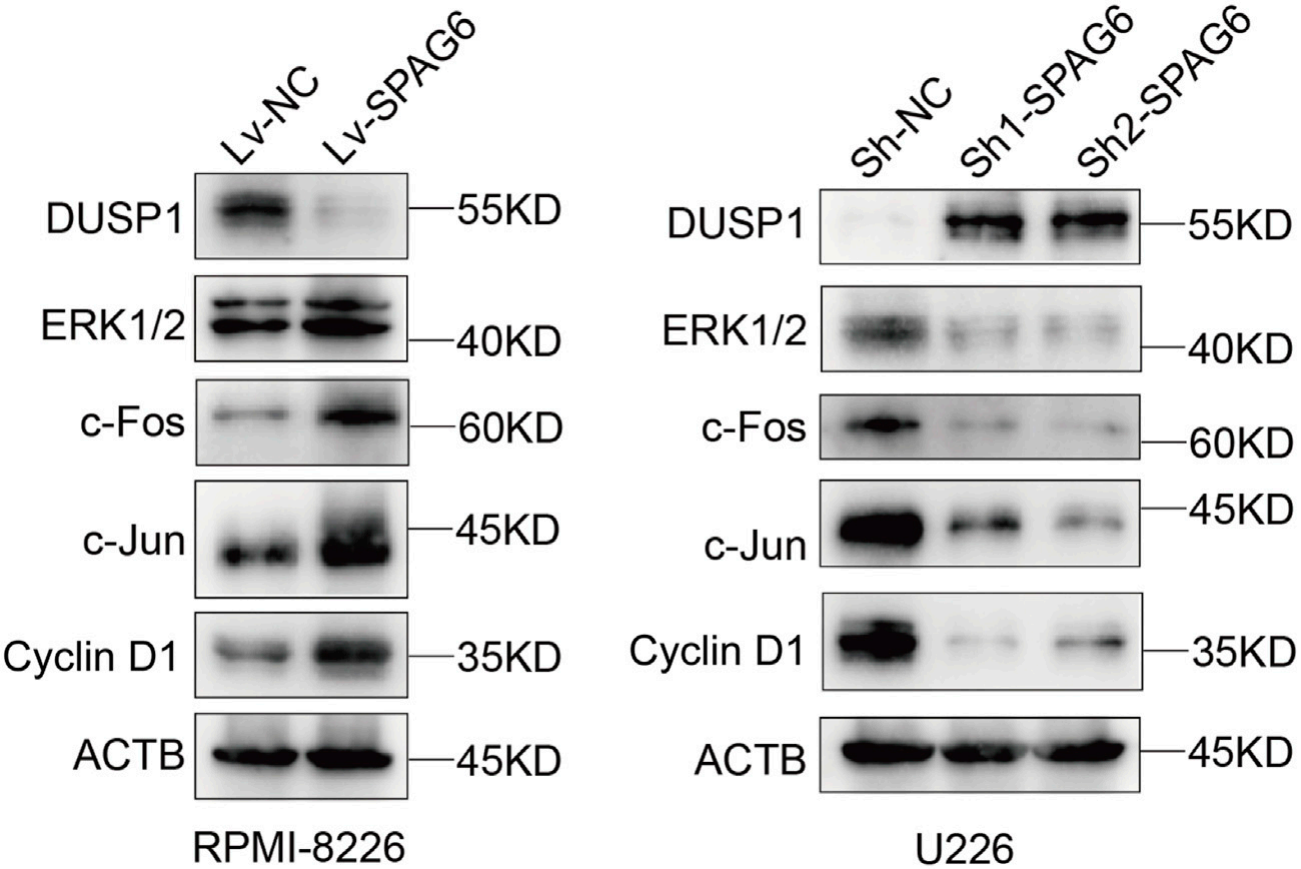
Western blotting was performed to evaluate the expression of proteins in the MAPK signaling pathway, including DUSP1, ERK 1/2, c-Fos, c-Jun, and Cyclin D 1, in response to changes in SPAG6 expression in RPMI-8226 and U266 cells.

DUSP1 functions as a key component of the MAPK/ERK signaling pathway, directly modulating downstream signaling events. Transcriptome sequencing further revealed that upon SPAG6 knockdown in U266 cells, the expression of DUSP1 was significantly upregulated, while the expression of upstream RAF genes in the MAPK/ERK signaling pathway exhibited no significant change. Therefore, we hypothesized that SPAG6 exerts its effects on the downstream signals of the MAPK/ERK pathway through its interaction with DUSP1. To validate this hypothesis, we conducted verification in two MM cell lines. Western blot analysis demonstrated that in RPMI-8226 cells, SPAG6 overexpression led to a notable downregulation of DUSP1 and correspondingly increased activation of downstream ERK pathway proteins, including ERK 1/2, c-Fos, c-Jun, and Cyclin D1. Conversely, in U266 cells, SPAG6 knockdown significantly upregulated DUSP1 expression, accompanied by reduced activation of these downstream proteins (Figure 8). Thus, we posit that the mechanism through which SPAG6 activates the MAPK signaling pathway via DUSP1 to regulate the biological functions of MM cells is depicted in Supplementary Figure S1.

## 4 Discussion

Plasma cell neoplasms include non-IgM monoclonal gammopathy of undetermined significance (MGUS), MM, extraosseous/extramedullary plasmacytoma (EMP), solitary plasmacytoma of bone, SBP) and monoclonal Ig deposition diseases (Campo et al., 2022). MM is the most commonly observed in clinical practice and involves complex genetic mechanisms, molecular changes lead to the clonal expansion and progression of plasma cells (Wallington-Beddoe et al., 2018). The emergence and extensive implementation of second-generation sequencing technology have enabled the uncovering of more molecular mechanisms underlying MM. The common mutated genes in MM include KRAS, NRAS, BRAF, TRAF, CYLD, LTB, ATM, DIS3, IRF4, EGR1, TP53, ATR, FAM46C, etc. Alterations in these molecules drive MM pathogenesis by regulating essential cellular processes in myeloma cells, including apoptosis, proliferation, migration, and autophagy (Boyle et al., 2021; Zhu et al., 2014).

SPAG6 was initially identified as a new human sperm antigen by screening human complementary DNA (cDNA) libraries using human serum containing high-titer anti-sperm antibodies and located at 10p11.2-p12 with a molecular size of 55.3kda (Neilson et al., 1999). Accumulating evidence indicates SPAG6 regulates oncogenic processes in multiple malignancies, with upregulated expression in lung squamous cell carcinoma correlating with tumor invasion (Wu et al., 2022). In hematological malignancy, SPAG6 expression was found to be elevated in patients with MDS than in healthy patients and could regulate the proliferation, cell cycle, and apoptosis of MDS cells (Bao et al., 2022; Jiang et al., 2019; Mu et al., 2022). Moreover, SPAG6 expression is elevated in *de novo* AML patients but normalized in those achieving complete remission post-treatment, while correlating with genetic signatures and regulating proliferation and migration of AML cells (Steinbach et al., 2006; Jiang et al., 2019; Mu et al., 2022). Furthermore, SPAG6 has been significantly upregulated in patients with MPN, with differential expression across various subtypes. Additionally, the forced expression of SPAG6 has been shown to induce proliferation and cell cycle progression in MPN cells (Ding et al., 2022). Another study conducted on a mouse model of Burkitt lymphoma confirmed that SPAG6 facilitated the proliferation of Burkitt lymphoma cells by activating the PTEN/PI3K/AKT signaling pathway (Zhang R. et al., 2020).

This study demonstrated SPAG6 upregulation in plasmacytoma tissues, and the IRS score in patients with multiple myeloma was significantly higher compared to those with solitary bone plasmacytoma. This suggests that SPAG6 expression may influence the phenotype of plasmacytoma and is associated with the progression of plasma cell disease. Furthermore, the expression of SPAG6 was found to be upregulated in the bone marrow of patients with MM, correlating with increased organ damage, a higher proportion of monoclonal plasma cells, and elevated serum calcium levels. This also indicates that SPAG6 may play a crucial role in the development of disease in patients with MM. Nevertheless, the present study is constrained by small sample size and short follow-up duration, necessitating enlargement of the cohort and prolongation of the observation period to enable further validation. *In vitro* experiment, the study demonstrated SPAG6 overexpression promoted the proliferation, migration of MM cells, and augmented cell resistance to apoptosis-inducing agents. Conversely, SPAG6 knockdown attenuated MM cell proliferation and migration while inducing apoptosis. Collectively, this study identified SPAG6 expression as a novel biomarker associated with MM clinical phenotypes and a regulator of MM cell biological behaviors.

Further analysis identified multiple SPAG6-correlated genes and suggested a potential interaction between SPAG6 and DUSP1 in activating the MAPK signaling pathway. The MAPK pathway is an intracellular signal transduction pathway that can regulate a variety of cellular functions, including cell proliferation, stress response, cell migration, and apoptosis (Rajkumar and Kumar, 2020; Simanshu et al., 2017; Degirmenci et al., 2020). The MAPK signaling pathway is critically involved in MM progression, with KRAS, NRAS, and BRAF mutations representing the most frequent genetic alterations driving disease progression (Manier et al., 2017). The four downstream MAPK pathways have been identified in eukaryotic cells: extracellular regulated protein kinases (ERK), c-Jun-N-terminal kinase/stress-activated protein kinase, p 38, and ERK5 signal transduction pathways. Ras protein is the upstream activator of the pathway and then activates RAF protein, MAPK/ERK kinase, and ERK, ultimately forming the Ras-Raf-MEK-ERK pathway (Xu et al., 2013). ERK protein is a member of the MAPK family and plays a role in signal transmission from extracellular to cells, including ERK1 and ERK2 (Müller et al., 2017). DUSP1 is the first member of the MAP kinase phosphatase (MKP) family, also known as MKP1, DUSP1/MKP1 is a dual-specificity phosphatase that regulates MAPK activity and plays a key role in tumor biology (Wancket et al., 2012). DUSP1 is 1,101 bp in size, can encode 367 amino acids, and specific targets ERK 1/2 in the MAPK signal pathway. Previous evidence indicates DUSP1/MKP1 overexpression in colon, bladder, and non-small cell lung cancers, where it modulates angiogenesis, invasion, and metastasis (Moncho-Amor et al., 2011). Conversely, in hepatocellular carcinoma, prostate cancer, and head/neck squamous cell carcinoma, DUSP1 exhibits significant downregulation as a tumor suppressor (Leelahavanichkul et al., 2014; Zhang et al., 2018; Du et al., 2020). DUSP1 exerts context-dependent effects on tumor cell biology, likely influenced by tumor microenvironments, while mediating distinct signaling pathways across malignancies. In prostate cancer, DUSP1 is known to dephosphorylate the JNK signaling pathway, suppressing tumor cell migration, invasion, and angiogenesis (Zhang et al., 2018). While in liver carcinoma, DUSP1 impacted the p38/MAPK pathway (Du et al., 2020). Recently, studies on endometrial cancer have revealed that DUSP1 and AP-1 exhibit a mutual regulatory relationship. DUSP1 can modulate the MAPK pathway by dephosphorylating ERK, thereby suppressing cell proliferation and facilitating cell apoptosis (Lei et al., 2023). This study identified a SPAG6-DUSP1 interaction in MM cells, where SPAG6 activates the MAPK/ERK pathway via downregulation, promoting downstream protein activation and cell proliferation/migration.

Nonetheless, this study has certain limitations, although existing evidence supports an association between SPAG6 and MM patients as well as pathological tissues, enlarging the sample size is essential for further validation. Moreover, due to time limitations, rescue experiments for intervening DUSP1 expression and the vitro studies were not conducted. However, we have formulated detailed plans for integrating these investigations into our future research agenda, specifically to further validate our findings and explore the underlying mechanisms in greater depth.

Generally, this study elucidates SPAG6’s role in MM, uncovering potential mechanisms contributing to MM pathogenesis while potentially informing the development of novel targeted therapies.

## Data Availability

The datasets presented in this study can be found in online repositories (https://osf.io/teq4u/). Further inquiries can be directed to the corresponding author.

## Supplementary material

SUPPLEMENTARY FIGURE S1 Schematic illustration of SPAG6-mediated regulation of multiple myeloma cell biology.

## Abbreviations

MM, multiple myeloma; SPAG6, sperm-associated antigen 6; CTAs, cancer/testis antigens; SBP, solitary plasmacytoma of bone; DUSP1, dual specificity phosphatase; MAPK, mitogen-activated protein kinase; ROC, receiver operating characteristic; AUC, area under the curve; UPD, uniparental disomy; MDS, myelodysplastic syndrome; MPN, myeloproliferative neoplasm; AML,acute leukemia; HAP, Human Protein Atlas; IMWG, International Myeloma Working Group; mSMART, Risk stratification adopts Mayo Stratification of Myeloma and Risk-Adapted Therapy; IHC, immunohistochemistry; IRS, immunoreactive score; MNCs, marrow mononuclear cells; STR, short tandem repeats; FBS, fetal bovine serum; RNAi, RNA interference; RNAi, RNA interference; Co-IP, Co-immunoprecipitation; CCK - 8, cell counting kit; EdU, 5-ethynyl-2′-deoxyuridine; MGUS, monoclonal gammopathy of undetermined significance; EMP, extraosseous/extramedullary plasmacytoma; cDNA, complementary DNA; ERK, extracellular regulated protein kinases; qPCR, quantitative PCR.
