# Supplementary figures and images for: SPAG6 Promotes Multiple Myeloma Through Activation of the MAPK/ERK Signaling Pathway
Source: Front Pharmacol. 2025 Jun 4;16:1572621. doi: 10.3389/fphar.2025.1572621 (PMC12174100; doi:10.3389/fphar.2025.1572621)

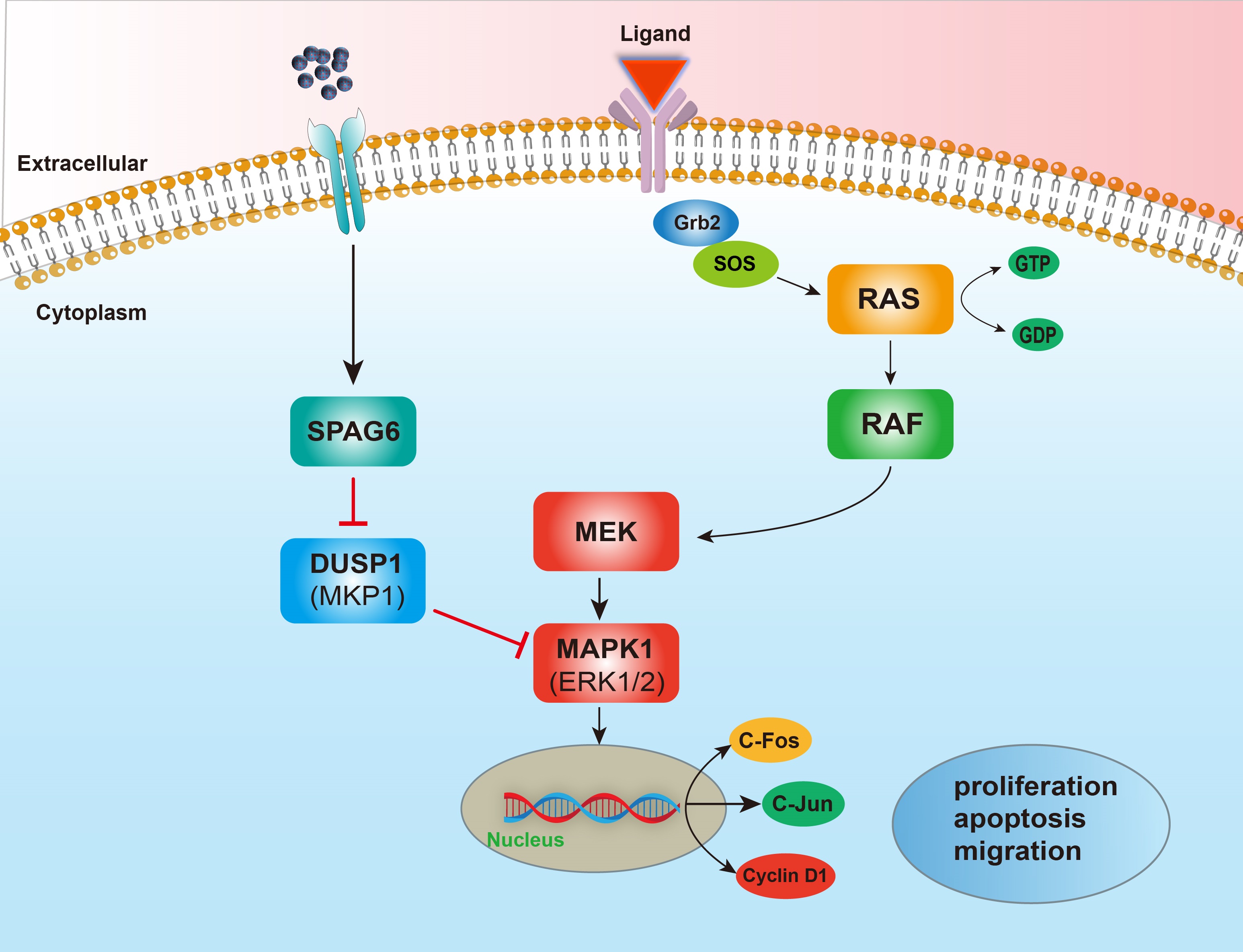

Supplement: Supplementary file 1 [file Image1.jpeg]
